# Antibody-mediated blockade of phosphatidylserine enhances the anti-tumor activity of targeted therapy and immune checkpoint inhibitors by affecting myeloid and lymphocyte populations in the tumor microenvironment

**DOI:** 10.1186/2051-1426-2-S3-P266

**Published:** 2014-11-06

**Authors:** Adam Yopp, Nikoletta Kallinteris, Xianming Huang, Joe Shan, Kerstin Menander, Bruce Freimark, Jeff Hutchins, Steve King, Dmitry I Gabrilovich, Rolf Brekken

**Affiliations:** 1 UTSW, USA; 2 Department of Clinical Affairs, Peregrine Pharmaceuticals Inc.,, Tustin, CA, USA; 3 Peregrine Pharmaceuticals, USA; 4 Peregrine Pharmaceuticals, Inc, Tustin, CA, USA; 5 The Wistar Institute, USA

## 

The underlying cause for the failure of immune checkpoint blockade is the overwhelming, persistent and multifocal immune suppression in the tumor microenvironment. This is due to the absence of pre-existing antitumor T_eff _because of the action of important upstream immune checkpoints that recruit immunosuppressive cytokines (e.g., TGF-beta and IL-10) and tumor infiltrating myeloid-derived suppressor cells (MDSCs), regulatory T cells (Tregs) and M2 macrophages that can occupy up to 50% of the tumor mass.

The membrane phospholipid, phosphatidylserine (PS), is an upstream immune checkpoint. In normal non-tumorigenic cells, PS is segregated to the inner leaflet of the plasma membrane but becomes externalized to the outer leaflet of the plasma membrane in cells in the tumor microenvironment. PS is recognized and bound by PS receptors on immune cells where it induces and maintains immune suppression. PS-targeting agents block PS-mediated immunosuppression by multifocal reprograming of the immune cells in the tumor microenvironment to support immune activation. Antibody-mediated PS blockade reduces the levels of MDSC, TGF-beta, and IL-10 and increases the levels of TNF-alpha and IL-12. PS blockade also re-polarizes tumor-associated macrophages (TAM's) from predominant M2 to predominant M1 phenotype, promotes the maturation of dendritic cells (DC's) and induces potent adaptive antitumor T cell immunity.

In a Phase II clinical study, immunohistochemical evaluation of HCC tumor tissues post combination treatment indicated an increase of immune infiltrates; raising the potential of a clinically meaningful anti-tumor immune response. Pre-clinically, we demonstrate that PS targeting agents enhance the anti-tumor activity of anti-CTLA-4 and anti-PD-1 antibodies in immunocompetent models of melanoma (B16 and K1735) and breast (EMT-6) cancer and that tumor growth inhibition correlates with an increase in the infiltration of activated T cells and myeloid cells and the induction of adaptive immunity. In summary, PS blockade in combination with targeted therapy and other immune checkpoint inhibitors promotes a robust, localized, anti-tumor response and represents a promising strategy to enhance cancer immunotherapy.

